# Fatores Associados à Ocorrência de Hipertensão Arterial em Trabalhadores da Indústria do Estado do Rio Grande do Sul, Brasil

**DOI:** 10.36660/abc.20190815

**Published:** 2021-06-10

**Authors:** Paula Brustolin Xavier, Anderson Garcez, Gabriela Herrmann Cibeira, Antonino Germano, Maria Teresa Anselmo Olinto

**Affiliations:** 1 Programa de Pós-Graduação em Saúde Coletiva Universidade do Vale do Rio dos Sinos São Leopoldo RS Brasil Programa de Pós-Graduação em Saúde Coletiva - Universidade do Vale do Rio dos Sinos (UNISINOS), São Leopoldo, RS - Brasil; 2 Área de Ciências da Vida Universidade do Oeste de Santa Catarina Joaçaba SC Brasil Área de Ciências da Vida - Universidade do Oeste de Santa Catarina (UNOESC), Joaçaba, SC - Brasil; 3 Programa de Pós-graduação em Ciências da Nutrição Universidade Federal de Ciências da Saúde de Porto Alegre Porto Alegre RS Brasil Programa de Pós-graduação em Ciências da Nutrição - Universidade Federal de Ciências da Saúde de Porto Alegre (UFCSPA), Porto Alegre, RS - Brasil; 4 Serviço Social da Indústria do Estado do Rio Grande do Sul Porto Alegre RS Brasil Serviço Social da Indústria do Estado do Rio Grande do Sul (SESI-RS), Porto Alegre, RS - Brasil; 5 Programa de Pós-graduação em Alimentação, Nutrição e Saúde Universidade Federal do Rio Grande do Sul Porto Alegre RS Brasil Programa de Pós-graduação em Alimentação, Nutrição e Saúde - Universidade Federal do Rio Grande do Sul (UFRGS), Porto Alegre, RS - Brasil

**Keywords:** Hipertensão, Fatores de Risco, Epidemiologia, Hereditariedade, Obesidade, Trabalhadores, Indústrias

## Abstract

**Fundamento:**

A hipertensão é um importante e persistente problema de saúde pública, sendo uma das principais causas de doenças cardiovasculares e mortalidade geral.

**Objetivos:**

Este estudo buscou verificar a prevalência e os fatores associados à hipertensão arterial sistêmica em trabalhadores da indústria do estado do Rio Grande do Sul, Brasil.

**Métodos:**

Trata-se de um estudo transversal com dados secundários de 20.792 industriários de 18 a 59 anos de idade. A presença de hipertensão arterial foi determinada a partir da pressão arterial sistólica ≥140mmHg e/ou pressão arterial diastólica ≥90mmHg, ou estar fazendo uso de medicação anti-hipertensiva. Os fatores investigados incluíram características demográficas, socioeconômicas, comportamentais, de estado nutricional e de história familiar. Regressão de Poisson foi utilizada na análise multivariável, adotando-se um p<0,05 como nível de significância. Todas as análises foram estratificadas por sexo.

**Resultados:**

A amostra incluiu 12.349 homens e 8.443 mulheres com média de idade geral de 32,8 anos (Desvio-padrão=9,8 anos). A prevalência de hipertensão foi de 10,3% (IC95%:9,8-10,7), sendo esta significativamente maior entre os homens do que entre as mulheres (10,9% vs 9,4%;p=0,001). A hipertensão mostrou-se associada à elevação da faixa etária, baixa escolaridade, viver com companheiro, ter sobrepeso ou obesidade, e ter pelo menos um parente com história de hipertensão para ambos os sexos. As mulheres com melhores condições socioeconômicas apresentaram menores prevalências de hipertensão.

**Conclusões:**

Os principais fatores associados à hipertensão arterial compreenderam características sociodemográficas, nutricionais e de história familiar. Ademais, as condições socioeconômicas demonstraram uma associação com a ocorrência de hipertensão, principalmente entre as mulheres.

## Introdução

A Hipertensão Arterial Sistêmica (HAS) é um importante problema de saúde pública, além de ser um fator de risco para doenças cardiovasculares.^1 - 4^ Estima-se que a prevalência de HAS na população mundial seja em torno de 22%.^5^ Na América Latina, o Brasil apresenta uma das maiores prevalências de HAS, com diferença significativa entre homens (26,7%) e mulheres (19,9%).^6^ Em trabalhadores da indústria, a prevalência de HAS diverge conforme a localização geográfica, sendo mais prevalente na região Nordeste (35,1%), seguido do Centro-Oeste (19%) e Sul do Brasil (19,8%).^7^

Potenciais fatores de risco para a ocorrência de HAS têm sido explorados em diferentes grupos populacionais de trabalhadores, evidenciando-se uma forte influência de fatores sociodemográficos, comportamentais e ocupacionais.^7 - 9^ A HAS tem sido associada principalmente aos hábitos comportamentais como o consumo de álcool e tabaco, dieta desequilibrada, inatividade física, além do possível efeito do histórico familiar da doença.^10^ Dessa forma, hábitos de vida não saudáveis também podem influenciar na ocorrência de outras doenças associadas a HAS, especialmente quando associados ao histórico familiar e presença de obesidade.^9^

Os fatores de risco cardiometabólicos normalmente são assintomáticos, e identificá-los precocemente permite que medidas e ações de prevenção possam ser estabelecidas.^11^ Além disso, os diferentes cenários ocupacionais e formas de gestão produtiva têm incorporado mudanças no cotidiano dos trabalhadores.^12^ Apesar de estudos prévios terem investigado a ocorrência de hipertensão entre trabalhadores da indústria no Brasil,^8 , 13^ não se identificaram estudos conduzidos no estado do Rio Grande do Sul, um dos estados mais industrializados do país. Dessa forma, o presente estudo buscou verificar a prevalência e investigar os fatores associados com a HAS em trabalhadores do setor da indústria do estado do Rio Grande do Sul, Brasil.

## Métodos

Estudo transversal com dados secundários do “Projeto Coração” do Serviço Social da Indústria do Rio Grande do Sul (SESI-RS), realizado no período de 2006 a 2009. A amostra contemplou trabalhadores adultos com 18 a 59 anos de idade e que atuavam em indústrias de pequeno (20 - 99 empregados), médio (100 - 499 empregados) e grande porte (> 500 empregados), distribuídas pelas regiões de maior industrialização do estado do Rio Grande do Sul. Embora o presente estudo tenha utilizado dados secundários, este foi submetido (CAAE: 90968018.9.0000.5344/2018) e aprovado pelo Comitê de Ética em Pesquisa da Universidade do Vale do Rio dos Sinos (Parecer n. 2.719.764). Dessa forma, a preservação do anonimato dos trabalhadores foi estabelecida junto ao termo de responsabilidade submetido à instituição-fonte dos dados.

### Amostragem

No sentido de garantir uma representatividade dos trabalhadores da indústria do Estado, e considerando a impossibilidade de relacionar todos os empregados, um processo de amostragem em duas etapas foi realizado. Na primeira, foi selecionada uma amostra aleatória simples das indústrias. Na segunda, obteve-se uma amostra de empregados registrados nas indústrias selecionadas na primeira etapa. A seleção das empresas foi realizada por meio de levantamento da sua localização (municípios) e número total de empregados, incluindo todas as atividades econômicas da Relação Anual de Informações Sociais (RAIS), ano-base 2004. Para determinar quais municípios deveriam ser incluídos na pesquisa, foi organizada uma lista contemplando os municípios que concentravam 80% dos empregos industriais do estado, distribuídos segundo o tamanho da empresa e Classificação Nacional de Atividades Econômicas (CNAEs). Por meio de escolha aleatória simples e estratificada segundo porte, foram sorteadas 145 empresas, contemplando indústrias do setor de alimentos e bebidas (CNAE 10 e 11), de produtos de couro (CNAE 15), de produtos metalmecânicos (CNAEs 24, 25, 28 e 29), e de fumo (CNAE 12). Esse processo visou contemplar uma amostra representativa em relação à população de empresas, considerando um nível de confiança de 95%, uma variância para o estimador na proporção de 0,25, e um erro máximo nas estimativas de proporções de 3,6 pontos percentuais. Posteriormente, obteve-se uma amostra proporcional ao número total de trabalhadores registrados nas empresas. Assim, ao final das duas etapas, contemplou-se a avaliação de 21.341 trabalhadores.

### Coleta de dados e instrumentos

A coleta de dados ocorreu por meio de entrevistas presenciais, durante o horário de trabalho e incluindo todos os turnos de trabalho existentes na empresa. Para garantir a padronização, todos os entrevistadores receberam treinamento sobre os procedimentos adequados para a coleta dos dados. Todas as entrevistas e avaliações foram realizadas dentro das dependências das empresas, sendo que os trabalhadores foram previamente liberados pelas empresas. Além disso, a adesão dos trabalhadores foi totalmente voluntária e consentida.

Foi utilizado um questionário padronizado, pré-codificado e pré-testado. O questionário contemplou informações demográficas, socioeconômicas, comportamentais, de estado nutricional, e de histórico familiar. As características demográficas investigadas foram: sexo, idade (faixas etárias de 10 anos), cor da pele (brancas e não-brancas) e situação conjugal (com e sem companheiro). Dentre as socioeconômicas, incluíram-se renda familiar mensal (estratificada em valores absolutos de reais) e escolaridade (estratificada em grau de ensino).

Quanto às características comportamentais, investigou-se tabagismo (fumante, não fumante e ex-fumante), considerando-se “fumante” o trabalhador que estivesse em uso de qualquer tipo ou quantidade de tabaco, diariamente, por ao menos seis meses. “Ex-fumante” foi definido como aquele que, tendo sido fumante, não tenha feito uso de tabaco nos últimos seis meses; e “não fumante” aquele que nunca fez uso de tabaco em qualquer período da vida. Já o consumo de álcool foi definido com base no consumo regular de qualquer tipo de bebida alcoólica em, no mínimo, uma vez por semana. Outra característica comportamental investigada foi a prática de atividade física regular, em que os participantes foram divididos entre ativos e inativos, considerando-se como atividade física regular aquelas praticadas de forma regular e constante.

O estado nutricional foi avaliado por meio do Índice de Massa Corporal – IMC, obtido pelas medidas de peso e altura, considerando a seguinte equação: peso (em quilogramas) dividido pela altura (em metros) ao quadrado. Foram classificados com sobrepeso todos os trabalhadores com IMC entre 25,0 e 29,9kg/m^2^, e com obesidade todos aqueles com 30,0 kg/m^2^ ou mais.^14^ Para a mensuração do peso corporal em quilogramas, foi utilizada balança digital previamente calibrada. A aferição da altura em centímetros foi realizada por meio de um estadiômetro móvel mantido sobre piso plano, sem rodapé e apoiado na parede. As medidas foram realizadas com os trabalhadores descalços, com roupas leves (sem casacos ou EPIs), totalmente eretos e com calcanhares unidos. O histórico familiar de hipertensão (mãe, pai e avós), foi relatado pelos participantes.

A variável “desfecho” foi obtida por meio da mensuração das pressões sistólicas e diastólicas, utilizando-se de estetoscópio e esfigmomanômetro do tipo aneroide, testados e calibrados pelo INMETRO (Instituto Nacional de Metrologia, Qualidade e Tecnologia). As aferições foram realizadas em duplicata, utilizando-se a média entre as duas medidas observadas. Todas as medidas foram obtidas no braço direito, com intervalo de 3 minutos, estando o indivíduo na posição sentada. Todas as mensurações foram executadas por estagiários de graduação em enfermagem. Estes foram previamente treinados, considerando a aplicação do protocolo de recomendações estabelecido pelo III Consenso Brasileiro de Hipertensão Arterial.^15^ As aferições foram realizadas, durante o horário de trabalho e previamente à aplicação dos questionários, em um espaço físico adequado e disponibilizado pelas empresas (afastado da área de produção e com baixos níveis de ruído).

A hipertensão arterial foi definida pela presença de pressão arterial sistólica ≥ 140mmHg e/ou pressão arterial diastólica ≥ 90mmHg, ou estar fazendo uso de medicação anti-hipertensiva regular ou esporádica.^4 , 16^ Trabalhadores com diagnóstico prévio de HAS e sem tratamento atual (uso de medicação) foram classificados com base no nível de pressão arterial mensurado no presente estudo.

### Análise estatística

Estatística descritiva foi utilizada para a distribuição geral da amostra e distribuição do desfecho em estudo, utilizando-se de frequências absoluta e relativa para as variáveis categóricas e média, com os seus respectivos desvios-padrões, para as variáveis numéricas. Para as análises brutas, entre a variável desfecho (HAS) e as variáveis de exposição (independentes), utilizou-se do teste do Qui-quadrado de Pearson para heterogeneidade de proporções (variáveis categóricas) ou de tendência linear (variáveis ordinais). Uma possível presença de colinearidade entre as variáveis de exposição foi avaliada por meio da associação entre elas.

Análise multivariável foi realizada por meio da Regressão de Poisson com variância robusta,^17^ considerando os valores de significância estatística obtidos por meio do teste Wald para heterogeneidade de proporções (variáveis categóricas) ou de tendência linear (variáveis ordinais). A análise multivariável baseou-se num modelo conceitual de determinação e das inter-relações das variáveis,^18^ considerando dois níveis hierárquicos de ajuste. No primeiro nível, foi realizada análise ajustada entre as características demográficas e socioeconômicas entre si. Já no segundo nível, foi realizada a análise ajustada incluindo as variáveis do primeiro nível com p<0,20, e as variáveis comportamentais, de estado nutricional e de história familiar. Todas as análises foram estratificadas por sexo, considerando a heterogeneidade da prevalência do desfecho (HAS). Todas as análises foram realizadas por meio do programa *Stata versão 12* (StataCorp LP, College Station, Texas, USA), considerando-se um nível de significância de 5% (p<0,05).

## Resultados

Dos 21.341 trabalhadores qualificados neste estudo, 489 (2,3%) foram classificados como perdas ou exclusões por falta de informações ou por estarem fora da faixa etária alvo, e em 60 trabalhadores (0,28%) não foi possível obter a medida de pressão arterial. Assim, um total de 20.792 trabalhadores com média de idade de 32,8 ± 9,8 anos foi incluída na análise final, considerando 12.349 homens (59,4%) e 8.443 (40,6%) mulheres, com médias de idade de 33,5 ± 10,1 anos e 31,9 ± 9,3 anos, respectivamente. Em relação aos setores da indústria, o presente estudo incluiu 4.356 trabalhadores do setor de alimentos e bebidas (20,9%), 9,692 do setor de produtos de couro (46,6%), 6,168 do setor de produtos metalmecânicos (29,7%), e 576 do setor de fumo (2,8%).

A Tabela 1 apresenta as características gerais da amostra para homens e mulheres e as respectivas prevalências de HAS conforme as variáveis investigadas. Na amostra geral, verificou-se uma prevalência de hipertensão arterial (HAS ≥ 140/90 mmHg ou tratamento) de 10,3% (IC95%: 9,8 - 10,7), sendo que 43,7% desses participantes não faziam uso de medicação anti-hipertensiva (n=931). A prevalência de HAS foi maior nos homens (10,9%; IC95%:10,3 - 11,4) do que entre as mulheres (9,4%; IC95%: 8,8 - 10,0; p=0,001), sendo que 24,3% dos homens e 25,8% das mulheres com idade acima de 40 anos apresentavam HAS. Verificou-se, também, que as mulheres com melhores condições socioeconômicas apresentaram menores prevalências de HAS quando comparado aos homens ( Tabela 1 ). Não foi identificada colinearidade entre nenhuma das variáveis independentes, incluindo idade e estado marital, assim como entre IMC, renda familiar e escolaridade.

**Tabela 1:** – Distribuição da amostra e prevalências de hipertensão arterial sistêmica (HAS≥140/90 mmHg ou tratamento) segundo características demográficas, socioeconômicas, comportamentais, estado nutricional e hereditárias, na amostra de homens e mulheres trabalhadores da indústria do estado do Rio Grande do Sul, RS, Brasil, 2006-2009. (N=20.792)

	Homens (12.349)			Mulheres (8.443)		
Características	n (%)	% HAS	p-valor	n (%)	% HAS	p-valor
Idade (anos)			<0,001			<0,001
18 a 29	5.180 (42,0)	2,6		3.925 (46,5)	2,0	
30 a 39	3.613 (29,2)	9,5		2.622 (31,1)	8,6	
40 a 49	2.593 (21,0)	20,1		1.522 (18,0)	22,6	
50 a 59	963 (7,8)	35,5		374 (4,4)	39,0	
Cor da pele			0,936			0,508
Branca	9.595 (77,7)	10,8		7.031 (83,3)	9,5	
Não Branca	2.754 (22,3)	10,9		1.412 (16,7)	8,9	
Situação conjugal			<0,001			<0,001
Sem companheiro	3.976 (32,2)	5,8		2.724 (32,3)	7,0	
Com companheiro	8.373 (67,8)	13,2		5.719 (67,7)	10,5	
Escolaridade			<0,001			<0,001
Fundamental incompleto	4.039 (32,7)	15,8		3.469 (41,1)	14,3	
Fundamental completo	2.932 (23,8)	8,6		2.060 (24,4)	7,5	
Médio completo	3.699 (29,9)	7,9		2.021 (24,0)	5,4	
Superior incompleto/completo	1.679 (13,6)	9,5		893 (10,6)	3,6	
Renda familiar mensal (reais)			0,004			0,002
≤ 800	2.973 (25,3)	10,6		2.410 (29,7)	10,6	
801 a 1.200	3.113 (26,4)	10,0		2.819 (34,7)	9,5	
1.201 a 1.800	2.518 (21,3)	10,0		1.578 (19,5)	9,9	
> 1.800	3.183 (27,0)	12,9		1.313 (16,2)	7,1	
Tabagismo			<0,001			0,014
Não fumante	9.051 (73,3)	9,8		7.153 (84,8)	9,2	
Ex-fumante	1.267 (10,3)	16,7		519 (6,1)	12,9	
Fumante	2.031 (16,4)	12,0		771 (9,1)	8,4	
Consumo de álcool			0,435			0,001
Não consome	7.650 (61,9)	10,7		7.483 (88,6)	9,8	
Consome	4.699 (38,1)	11,1		960 (11,4)	6,6	
Atividade física			<0,001			0,324
Ativo	4.419 (36,0)	8,9		2.054 (24,5)	9,9	
Inativo	7.860 (64,0)	11,9		6.347 (75,5)	9,2	
Estado nutricional (IMC)			<0,001			<0,001
Normal (< 25 kg/m^2^	5.664 (46,0)	4,7		4.538 (53,9)	3,6	
Sobrepeso (25 a 29,9 kg/m^2^)	5.014 (40,6)	13,0		2.505 (29,8)	11,9	
Obeso (≥ 30 kg/m^2^)	1.660 (13,5)	25,4		1.380 (16,3)	24,0	
Presença de hipertensão na família			<0,001			<0,001
Não	6.442 (52,2)	7,8		3.477 (41,2)	4,8	
Mãe ou Pai	4.987 (40,5)	12,5		3.950 (46,8)	10,3	
Mãe e Pai	674 (5,5)	22,9		657 (7,8)	22,2	
Mãe, Pai e Avós	226 (1,8)	25,7		349 (4,1)	21,2	

*IMC: índice de massa corporal. p-valor para teste do Qui-quadrado para heterogeneidade de proporções (variáveis categóricas) ou tendência linear (variáveis ordinais)*

A Tabela 2 apresenta as razões de prevalências brutas e ajustadas, estratificadas entre homens e mulheres, para a ocorrência de HAS conforme os fatores investigados. Após análise ajustada, a prevalência de HAS mostrou-se associada à elevação da faixa etária, baixa escolaridade, viver com companheiro, ter sobrepeso ou obesidade, e ter pelo menos um familiar com história de hipertensão. Quanto aos fatores socioeconômicos, a menor escolaridade manteve-se associada com maiores prevalências de HAS apenas entre as mulheres. Associação diretamente proporcional foi observada entre estado nutricional e HAS em ambos os sexos. Ainda, a prevalência de HAS foi direta e significativamente maior entre aqueles que tinham um dos pais, ou ambos os pais e mais os avós, com histórico de hipertensão. Embora renda familiar, tabagismo, consumo de álcool e prática de atividade física tenham apresentado uma tendência de associação na análise bruta, estas foram contraditórias e todas perderam significância após o ajuste ( Tabela 2 ).

**Tabela 2:** – Razões de prevalência (RP) brutas e ajustadas de hipertensão arterial sistêmica (HAS≥140/90 mmHg ou tratamento) segundo características demográficas, socioeconômicas, comportamentais, estado nutricional e hereditárias, na amostra de homens e mulheres trabalhadores da indústria do estado do Rio Grande do Sul, RS, Brasil, 2006-2009. (N=20.792)

		Homens (12.349)		Mulheres (8.443)	
	Características	RP Bruta*	RP Ajustada†	RP Bruta*	RP Ajustada†
**Primeiro Nível**	Idade (anos)	p<0,001	p<0,001	p<0,001	p<0,001
	18 a 29	1	1	1	1
	30 a 39	3,66 (3,01-4,45)	3,43 (2,78-4,21)	4,32 (3,35-5,56)	3,79 (2,93-4,90)
	40 a 49	7,78 (6,48-9,35)	7,13 (5,84-8,71)	11,37 (9,00-14,44)	9,85 (7,70-12,60)
	50 a 59	13,7 (11,38-16,56)	12,09 (9,81-14,90)	19,64 (15,24-25,31)	17,28 (13,29-22,47)
	Cor da pele	p=0,936	-	p=0,509	-
	Branca	1		1	
	Não Branca	1,01 (0,89-1,13)		0,95 (0,78-1,13)	
	Situação conjugal	p<0,001	p=0,028	p<0,001	p<0,001
	Sem companheiro	1	1	1	1
	Com companheiro	2,27 (1,98-2,60)	1,17 (1,02-1,35)	1,50 (1,28-1,76)	1,28 (1,09-1,51)
	Escolaridade	p<0,001	p=0,182	p<0,001	p=0,001
	Fundamental incompleto	1,67 (1,41-1,96)	1,05 (0,87-1,26)	4,00 (2,82-5,67)	1,58 (1,08-2,29)
	Fundamental completo	0,91 (0,75-1,10)	0,84 (0,70-1,03)	2,10 (1,45-3,05)	1,46 (1,00-2,14)
	Médio completo	0,83 (0,69-1,00)	0,88 (0,73-1,06)	1,51 (1,02-2,21)	1,30 (0,87-1,92)
	Superior incompleto/completo	1	1	1	1
	Renda familiar mensal (reais)	p=0,005	p=0,701	p=0,002	p=0,055
	≤ 800	0,83 (0,72-0,95)	0,96 (0,83-1,12)	1,50 (1,19-1,88)	1,30 (1,02-1,66)
	801 a 1.200	0,77 (0,67-0,89)	0,89 (0,77-1,03)	1,35 (1,07-1,69)	1,20 (0,95-1,51)
	1.201 a 1.800	0,78 (0,67-0,90)	0,89 (0,76-1,03)	1,40 (1,09-1,79)	1,16 (0,91-1,49)
	> 1.800	1	1	1	1
**Segundo Nível**	Tabagismo	p<0,001	p=0,262	p=0,014	p=0,133
	Não fumante	1	1	1	1
	Ex-fumante	1,70 (1,48-1,96)	1,03 (0,91-1,18)	1,40 (1,10-1,77)	0,96 (0,77-1,20)
	Fumante	1,23 (1,08-1,40)	1,08 (0,95-1,23)	0,91 (0,71-1,16)	0,83 (0,67-1,05)
	Consumo de álcool	p=0,435	-	p=0,002	p=0,824
	Não consome	1		1	1
	Consome	1,04 (0,94-1,16)		0,67 (0,52-0,86)	0,98 (0,77-1,25)
	Atividade física	p<0,001	p=0,361	p=0,323	-
	Ativo	1	1	1	
	Inativo	1,35 (1,20-1,51)	1,04 (0,94-1,16)	0,93 (0,80-1,08)	
	Estado nutricional (IMC)	p<0,001	p<0,001	p<0,001	p<0,001
	Normal (< 25 kg/m^2^)	1	1	1	1
	Sobrepeso (25 a 29,9 kg/m^2^)	2,78 (2,42-3,18)	1,94 (1,69-2,22)	3,34 (2,78-4,02)	2,04 (1,69-2,45)
	Obeso (≥ 30 kg/m^2^)	5,43 (4,71-6,27)	3,36 (2,90-3,89)	6,76 (5,66-8,08)	3,60 (2,98-4,36)
	Presença de HAS na família	p<0,001	p<0,001	p<0,001	p<0,001
	Não	1	1	1	1
	Mãe ou Pai	1,61 (1,44-1,80)	1,49 (1,34-1,65)	2,17 (1,82-2,58)	1,78 (1,50-2,10)
	Mãe e Pai	2,94 (2,50-3,46)	2,16 (1,86-2,52)	4,68 (3,81-5,76)	2,77 (2,27-3,38)
	Mãe, Pai e Avós	3,31 (2,61-4,19)	2,95 (2,40-3,62)	4,47 (3,48-5,74)	3,22 (2,54-4,07)

* p-valor para teste Wald para heterogeneidade de proporções (variáveis categóricas) ou tendência linear (variáveis ordinais) obtido por meio da regressão de Poisson com variância robusta; † Análise ajustada por modelo multivariável, incluindo as variáveis com p<0,20 na análise bruta. Primeiro Nível: ajuste entre as variáveis sociodemográficas; Segundo Nível: ajuste entre as variáveis do primeiro nível com p<0,20 mais as variáveis comportamentais, estado nutricional e hereditariedade.

## Discussão

Nossos achados apontaram que fatores sociodemográficos, nutricionais e de história familiar estão associados com a ocorrência de HAS nos trabalhadores da indústria. Ademais, identificou-se que a prevalência de HAS aumentou conforme o avanço da idade e situação conjugal, em ambos os sexos. Além disso, as condições socioeconômicas desfavoráveis se associaram com a presença de HAS, principalmente entre as mulheres.

A prevalência de HAS verificada neste estudo ficou abaixo do verificado em estudos conduzidos com trabalhadores da indústria no Brasil.^8 , 13^ Essa variabilidade pode ser potencialmente explicada pelo período de coleta de dados do presente estudo (2006 a 2009), pela diversidade de critérios utilizados para definir a presença de HAS, além do tipo de aparelho e momento de aferição da pressão arterial sistêmica, dificultando as comparações entre os estudos.^19^ Ademais, os trabalhadores homens apresentaram uma maior prevalência de HAS, quando comparados às mulheres, convergindo com um estudo prévio conduzido com trabalhadores da indústria.^7^

Embora estudos transversais não permitam estabelecer relações de causa-efeito, é possível considerar que as alterações fisiológicas relacionadas ao avanço da idade podem elevar os riscos no sistema cardiovascular.^1 , 20 , 21^ Neste estudo, a prevalência de HAS foi significativamente maior entre os trabalhadores com 40 anos ou mais de idade. Achado semelhante foi observado em um estudo conduzido com trabalhadores do setor de metalúrgica e siderúrgica,^13^ evidenciando que o trabalhador acima de quarenta anos é uma prioridade para ações de prevenção e intervenção para HAS.

Trabalhadores que relataram viver com companheiro(a) apresentaram uma maior prevalência de HAS. Achado semelhante foi previamente observado, em que viver com companheiro(a) esteve associado a presença de outras morbidades e HAS.^22^ Já ao analisar a influência da escolaridade na HAS, nosso estudo identificou que as mulheres com menor escolaridade apresentaram uma maior probabilidade para HAS, assemelhando-se a achados prévios em amostras de trabalhadores.^23 , 24^ Embora tenha-se buscado identificar diferenças entre os potencias fatores de risco para HAS entre trabalhadores de ambos os sexos, destaca-se que a suscetibilidade biológica relacionada ao sexo ou a diferenças de gênero, seja desvinculada dos riscos de saúde inerentes ao processo de trabalho para ambos os sexos.^25^ Contudo, cabe destacar que as mulheres muitas vezes ocupam os cargos de menor prestígio e com menores salários nas empresas,^26^ aspectos que podem refletir nas maiores prevalências de HAS em mulheres de menor posição socioeconômica.

Neste estudo, os trabalhadores com sobrepeso ou obesidade apresentaram uma probabilidade de duas a três vezes maior de ter HAS. Estudos prévios corroboram uma associação significativa entre estado nutricional e HAS, incluindo estudos conduzidos com trabalhadores.^12 , 20 , 27 , 28^ Esse achado demonstra uma possível relação entre o aumento da prevalência da HAS com a transição nutricional da população.^6 , 28^ Já no que se refere ao histórico familiar de HAS, verificamos uma prevalência de HAS significativamente maior entre aqueles trabalhadores que reportaram história familiar pregressa de HAS em pai, mãe ou avós. Estudos prévios sobre o tema, demonstram uma importância significativa da carga hereditária para o desenvolvimento da HAS.^29 , 30^

Destaca-se como principal ponto positivo deste estudo o seu tamanho amostral, incluindo trabalhadores de diferentes setores da indústria do estado do Rio Grande do Sul. O presente estudo adotou uma coleta padronizada dos dados, assim como todos os procedimentos necessários para a adequada aferição da pressão arterial. Ressalta-se, ainda, que procurou-se explorar os potenciais fatores associados à ocorrência de HAS, considerando a adoção de um modelo de análise multivariável. Contudo, os achados deste estudo devem ser interpretados considerando-se algumas limitações. Trata-se de um estudo com o uso de dados secundários, coletados entre os anos de 2006 e 2009, não incluindo a coleta de variáveis ocupacionais. Por se tratar de um estudo com desenho transversal, este pode estar sujeito a presença de causalidade reversa entre as associações investigadas. A presença do viés do trabalhador saudável ou sobrevivente também não pode ser totalmente descartada, uma vez que se consideraram no estudo apenas os trabalhadores ativos, ou seja, aqueles que estavam exercendo suas atividades laborais no momento de aplicação do estudo, excluindo os que se encontravam afastados por algum fator de saúde, por exemplo. Outra limitação do estudo deve-se à falta de uma análise de variabilidade intra e interobservador para as mensurações das pressões sistólicas e diastólicas obtidas. Por fim, a realização de análises por setores da indústria, assim como possíveis diferenças sociodemográficas dentre os setores, ficaram prejudicadas em decorrência da grande heterogeneidade do número de trabalhadores investigados em cada setor.

Embora a prevalência de HAS encontrada em nosso estudo tenha sido relativamente baixa, observou-se que a ocorrência de HAS em trabalhadores da indústria é influenciada por fatores sociodemográficos, nutricionais e de história familiar. Contudo, é pertinente que outros estudos sejam realizados, tendo como objetivo identificar os potenciais fatores de risco ocupacionais (tipo de ocupação; turno de trabalho; tempo de serviço; presença de programa alimentar no trabalho; entre outros) associados a HAS, visando a medidas e ações específicas voltadas para a ocorrência de doenças crônicas não transmissíveis, e focadas na saúde integral do trabalhador.

## Conclusões

Nossos achados, na amostra estudada, apontaram que fatores sociodemográficos, nutricionais e de história familiar estão associados à ocorrência de HAS. Identificou-se que a prevalência de HAS aumentou conforme o avanço da idade e situação conjugal, em ambos os sexos. De maneira semelhante, as condições socioeconômicas desfavoráveis se associaram à presença de HAS, principalmente entre as mulheres. Tal evidência pode contribuir na formulação de intervenções efetivas para a prevenção da HAS junto às políticas voltadas à saúde do trabalhador.
