# Supplementary material for: Fatores Associados à Ocorrência de Hipertensão Arterial em Trabalhadores da Indústria do Estado do Rio Grande do Sul, Brasil
Source: Arq Bras Cardiol. 2021 Jun 10;117(3):484–91. [Article in Portuguese] doi: 10.36660/abc.20190815 (PMC8462968; doi:10.36660/abc.20190815)
Supplement: Translation

# Translation: Factors Associated with the Occurrence of Arterial Hypertension in Industry Workers of State of Rio Grande do Sul, Brazil

**Authors:** Paula Brustolin Xavier, Anderson Garcez, Gabriela Herrmann Cibeira, Antonino Germano, Maria Teresa Anselmo Olinto

**Affiliations:** 1 Brazil Programa de Pós-Graduação em Saúde Coletiva - Universidade do Vale do Rio dos Sinos (UNISINOS), São Leopoldo, RS - Brazil; 2 Brazil Área de Ciências da Vida - Universidade do Oeste de Santa Catarina (UNOESC), Joaçaba, SC - Brazil; 3 Brazil Programa de Pós-graduação em Ciências da Nutrição - Universidade Federal de Ciências da Saúde de Porto Alegre (UFCSPA), Porto Alegre, RS - Brazil; 4 Brazil Serviço Social da Indústria do Estado do Rio Grande do Sul (SESI-RS), Porto Alegre, RS - Brazil; 5 Brazil Programa de Pós-graduação em Alimentação, Nutrição e Saúde - Universidade Federal do Rio Grande do Sul (UFRGS), Porto Alegre, RS - Brazil

**Keywords:** Hypertension, Risk Factors, Epidemiology, Heredity, Obesity, Workers, Industry

## Abstract

**Background:**

Hypertension is a serious and persistent public health problem and is one of the main causes of cardiovascular diseases and general mortality.

**Objectives:**

This study aimed to verify the prevalence and factors associated with systemic arterial hypertension in workers from the state of Rio Grande do Sul, Brazil.

**Methods:**

This is a cross-sectional study using the secondary data from 20,792 industry workers from 18 to 59 years of age. The presence of arterial hypertension was determined from systolic blood pressure ≥ 140mmHg and/or diastolic blood pressure ≥ 90mmHg or taking antihypertensive medication. Factors investigated included demographic, socioeconomic, behavioral, nutritional status, and family history characteristics. Poisson regression was used in multivariate analysis, adopting a significance level of p<0.05. All analyses were stratified by sex.

**Results:**

The sample included 12,349 men and 8,443 women with a mean age of 32.8 years (Standard Deviation = 9.8). The prevalence of arterial hypertension was 10.3% (95% CI: 9.8-10.7), which was significantly higher in men than in women (10.9% vs 9.4%; p = 0.001). Arterial hypertension was associated with increased age, a low level of education, living with a partner, being overweight or obese, and having at least one relative with a history of hypertension for both sexes. Women with better socioeconomic conditions presented a lower prevalence of hypertension.

**Conclusions:**

The main factors associated with hypertension included sociodemographic, nutritional, and family history characteristics. In addition, socioeconomic conditions showed an association with the occurrence of hypertension, especially among women.

## Introduction

Systemic Arterial Hypertension (SAH) is a serious problem in public health, as well as a risk factor for cardiovascular diseases.^1 - 4^ It is estimated that the prevalence of SAH in the world population is approximately 22%.^5^ In Latin America, Brazil presents one of the highest prevalences of SAH, with a significant difference between men (26.7%) and women (19.9%).^6^ Among industry workers, the prevalence of SAH varies according to the geographic location and tends to be more prevalent in the Northeast region (35.1%), followed by the Midwest (19%) and the South (19.8%) regions of Brazil.^7^

Potential risk factors for the occurrence of SAH have been explored in different worker population groups, showing a heavy influence of sociodemographic, behavioral, and occupational factors.^7 - 9^ SAH has mainly been associated with behavioral habits, such as the consumption of alcohol and tobacco; dysfunctional diet; the lack of physical exercise; as well as possible effects of the family history of the disease.^10^ In this sense, unhealthy lifestyles can also influence the occurrence of other diseases associated with SAH, especially when related to family history and the presence of obesity.^9^

Cardiometabolic risk factors are normally asymptomatic and their early identification allows for preventive measures and actions to be established.^11^ In addition, the different occupational scenarios and forms of productive management have incorporated changes in workers’ daily routines.^12^ Although prior studies have investigated the occurrence of hypertension among industry workers in Brazil,^8 , 13^ no studies have been conducted in the state of Rio Grande do Sul, one of the most industrialized states of the country. In this light, the present study seeks to verify the prevalence and investigate the factors associated with SAH in industry workers from the state of Rio Grande do Sul.

## Methods

This is a cross-sectional study, using secondary data from the “Heart Project” (“Projeto Coração”) of the Social Services of the Industrial Sector of the state of Rio Grande do Sul (SESI-RS, in Portuguese), conducted from 2006 to 2009. The sample contemplated adult workers of 18 to 59 years of age and who have worked in small (20 – 99 employees), mid-sized (100 – 499 employees), and large (> 500 employees) industries, distributed throughout the main industrialized regions of the state of Rio Grande do Sul. Although this study used secondary data, it was submitted to (CAAE: 90968018.9.0000.5344/2018) and approved by the Research Ethics Committee of Universage do Vale do Rio dos Sinos (logged under protocol number 2.719.764). In this manner, the anonymity of the workers was preserved through a statement of responsibility submitted to the institution-data source.

### Sample

In an attempt to guarantee the representativeness of the state’s industry workers, and considering the impossibility of including all employees, a sampling process was conducted in two stages. In the first stage, a simple, random sample was selected from the industries. In the second stage, a sample of the workers registered from the industries in the first stage was obtained. The selection of the companies was carried out through a survey of their location (municipalities) and the total number of employees, including all economic activities of the Annual Report on Social Information (RAIS, in Portuguese) from 2004. To determine which municipalities should be included in the study, a list was made, contemplating the municipalities in which 80% of the industry workers of the state were concentrated, distributed according to the company size and the National Classification of Economic Activities (CNAEs, in Portuguese). Through this simple and stratified random selection, according to company size, 145 companies were selected, contemplating industries from the sectors of food and beverage (CNAE 10 and 11), leather products (CNAE 15), metal-mechanic products (CNAEs 24, 25, 28 e 29), and tobacco (CNAE 12). This process sought to contemplate a representative sample related to the population of companies, considering a 95% confidence interval (95% CI), a variance for the proportion estimator of 0.25, and a maximum error in the proportion estimates of 3.6 percentage points. This study then obtained a sample proportion of the total number of employees registered in the companies. Therefore, at the end of the two stages, the assessment of 21,341 workers was contemplated.

### Sample collection and instruments

The sample collection occurred by means of face-to-face interviews during working hours and including all of the company’s work shifts. To guarantee standardization, all of the interviewees received training on the proper procedures for data collection. All of the interviews and assessments were conducted on company grounds. All workers had received prior permission by the companies, and the adherence of the workers to this study was fully voluntary and consented.

A standardized, pre-coded, and pre-tested questionnaire was used. The questionnaire contemplated demographic, socioeconomic, behavioral, nutritional, and hereditary information. The demographic characteristics investigated in this study were: sex, age (10-year age groups), skin color (white and non-white), and marital status (with or without partner). The socioeconomic characteristics included: monthly family income (stratified in absolute values of Brazilian Reals) and education (stratified in level of education). As regards the behavioral characteristics, this study investigated smoking (smoker, non-smoker, or ex-smoker), considering a “smoker” to be the worker who uses any type or quantity of tobacco, daily, for at least six months. “Ex-smoker” was defined as those who have been a smoker and have not used tobacco in the last six months; “non-smoker” was defined as those who had never used tobacco at any time in their lives. The consumption of alcohol, by contrast, was defined based on the regular consumption of any type of alcoholic beverage at least once a week.

As regards the regular practice of physical activity (active and inactive), this study considered regular physical activity as practices performed in a regular and constant manner. The nutritional state was evaluated by means of the Body Mass Index (BMI), obtained by measuring one’s weight and height, considering the following equation: weight (in kilograms) divided by height (in meters) squared. All workers with a BMI of between 25.0 and 29.9kg/m^2^ were classified as overweight, while those with a BMI of 30.0 kg/m^2^ or more were classified as obese.^14^ To measure the body weight in kilograms, a previously calibrated digital scale was used. The measurement of height in centimeters was carried out by using a mobile stadiometer, kept on a flat plane, with no baseboard, and propped up on a wall. The measurements of the workers were taken without shoes, with light clothes (without coats or PPEs), totally erect, and with their heels together. The family history of hypertension (mother, father, and grandparents) was reported by the participants.

The outcome variable was obtained by measuring the systolic and diastolic blood pressures, using a stethoscope and an aneroid sphygmomanometer, tested and calibrated by the National Institute of Metrology Standardization and Industrial Quality (INMETRO). The measurements were carried out in duplicate, using the average of the two observed measurements. All of the measurements were taken on the right arm, at an interval of three minutes, with the individual in a sitting position. All measurements were performed by undergraduate interns in nursing. These interns had been previously trained, considering the application of the protocol of recommendations established by the Third Brazilian Guideline on Arterial Hypertension.^15^ The measurements were performed during working hours and before the application of the questionnaires, in an adequate physical space made available by the companies (apart from the production area and with low noise levels).

The presence of hypertension was determined by the presence of systolic arterial blood pressure ≥ 140mmHg and/or diastolic arterial blood pressure ≥ 90mmHg or taking antihypertensive medication, regularly or sporadically.^4 , 16^ Workers with a prior diagnosis of SAH and without current treatment (use of medication) were classified based on the worker’s blood pressure measured in the present study.

### Statistical analysis

Descriptive statistics was used for the general distribution of the sample and the distribution of the outcome under study, using absolute and relative frequencies for the categorical and average variables, with their respective standard deviations, for the numerical variables. For the gross analysis, between the outcome variable (SAH) and their exposure variables (independent), Pearson’s chi-squared text was used for the heterogeneity of the proportions (categorical variables) or of linear trend (ordinal variables). A possible presence of collinearity between the exposure variables was assessed through their association.

Multivariate analysis was performed by means of Poisson Regression with robust variance,^17^ considering the values of statistical significance obtained through the Wald test for the heterogeneity of proportions (categorical variables) or of linear trend (ordinal variables). The multivariate analysis was based on the conceptual model of determination and interactions among variables,^18^ considering two hierarchical levels of adjustment. At the first level, the adjusted analysis was performed between the demographic and socioeconomic characteristics themselves. By contrast, at the second level, the adjusted analysis was performed, including the variables of the first level with p<0.20 and the behavioral, nutritional, and family history variables. All of the analyses were stratified by sex, considering the heterogeneity of the outcome prevalence (SAH), and were conducted using the Stata program, version 12 (StataCorp LP, College Station, Texas, USA), considering a significance level of 5% (p<0.05).

## Results

Of the 21,341 workers eligible in this study, 489 (2.3%) were classified as losses or exclusions, as they packed information or were outside of the age group target, while for 60 workers (0.28%), it was not possible to measure their blood pressure. Thus, a total of 20,792 workers, with an average age of 32.8 ± 9.8 years, was included in the final analysis, considering 12,349 men (59.4%) and 9,443 women (40.6%), with average ages of 33.5 ± 10.1 years and 31.9 ± 9.3 years, respectively. Regarding the industrial sectors, the present study included 4,356 workers from the food and beverage sector (20.9%), 9,692 from the leather products sector (46.6%), 6,168 from the metal-mechanic sector (29.7%), and 576 from the tobacco sector (2.8%).

Table 1 presents the general characteristics of the sample for men and women and the respective prevalence of SAH, according to the investigated variables. In the general sample, a prevalence of hypertension (SAH ≥ 140/90 mmHg or treatment) of 10.3% was found (95% CI: 9,8 - 10,7), given that 43.7% were not taking antihypertensive medication (n=931). The prevalence of SAH was greater in men (10.9%; 95% CI: 10.3 – 11.4) than in women (9.4%; 95% CI: 8.8 – 10.0; p=0.001), given that 24.3% of the men and 25.8% of the women, aged 40 years and over, presented SAH. This study also found that the women with better socioeconomic conditions presented a higher prevalence of SAH when compared to men ( Table 1 ). No collinearity was identified between any of the independent variables, including age and marital status, as well as between BMI, family income, and level of education.

**Table 1:** – Distribution of the sample and prevalence of systemic arterial hypertension (SAH≥140/90 mmHg or treatment) according to demographic, socioeconomic, behavioral, nutritional state, and hereditary characteristics in the sample of male and female industry workers in the state of Rio Grande do Sul, RS, Brazil, 2006-2009. (N=20.792)

Characteristics	Men (12,349)			Women (8,443)		
	n (%)	% SAH	p-value	n (%)	% SAH	p-value
Age (years)			<0.001			<0.001
18 to 29	5,180 (42.0)	2.6		3,925 (46.5)	2.0	
30 to 39	3,613 (29.2)	9.5		2,622 (31.1)	8.6	
40 to 49	2,593 (21.0)	20.1		1,522 (18.0)	22.6	
50 to 59	963 (7.8)	35.5		374 (4.4)	39.0	
Skin color			0.936			0.508
White	9,595 (77.7)	10.8		7,031 (83.3)	9.5	
Não White	2,754 (22.3)	10.9		1,412 (16.7)	8.9	
Marital status			<0.001			<0.001
Without partner	3,976 (32.2)	5.8		2,724 (32.3)	7.0	
With partner	8,373 (67.8)	13.2		5,719 (67.7)	10.5	
Education			<0.001			<0.001
Incomplete elementary	4,039 (32.7)	15.8		3,469 (41.1)	14.3	
Complete elementary	2,932 (23.8)	8.6		2,060 (24.4)	7.5	
Complete high school	3,699 (29.9)	7.9		2,021 (24.0)	5.4	
Incomplete/complete higher education	1,679 (13.6)	9.5		893 (10.6)	3.6	
Monthly Family income (Brazilian reals)			0.004			0,002
≤ 800	2,973 (25.3)	10.6		2,410 (29.7)	10.6	
801 to 1,200	3,113 (26.4)	10.0		2,819 (34.7)	9.5	
1,201 to 1,800	2,518 (21.3)	10.0		1,578 (19.5)	9.9	
> 1,800	3,183 (27.0)	12.9		1,313 (16.2)	7.1	
Smoking			<0.001			0.014
Non-smoker	9,051 (73.3)	9.8		7,153 (84.8)	9.2	
Ex-smoker	1,267 (10.3)	16.7		519 (6.1)	12.9	
Smoker	2,031 (16.4)	12.0		771 (9.1)	8.4	
Alcohol consumption			0.435			0.001
Does not consume	7,650 (61.9)	10.7		7,483 (88.6)	9.8	
Consumes	4,699 (38.1)	11.1		960 (11.4)	6.6	
Physical Activity			<0.001			0.324
Active	4,419 (36.0)	8.9		2,054 (24.5)	9.9	
Inactive	7,860 (64.0)	11.9		6,347 (75.5)	9.2	
Nutricional State (BMI)			<0.001			<0.001
Normal (< 25 kg/m^2^	5,664 (46.0)	4.7		4,538 (53.9)	3.6	
Overweight (25 to 29.9 kg/m^2^)	5.014 (40.6)	13.0		2,505 (29.8)	11.9	
Obese (≥ 30 kg/m^2^)	1,660 (13.5)	25.4		1,380 (16.3)	24.0	
Presence of hypertension in the family			<0.001			<0.001
No	6,442 (52.2)	7.8		3,477 (41.2)	4.8	
Mother or Father	4,987 (40.5)	12.5		3,950 (46.8)	10.3	
Mother and Father	674 (5.5)	22.9		657 (7.8)	22.2	
Mother, Father, and Grandparents	226 (1.8)	25.7		349 (4.1)	21.2	

*BMI: body mass index. P-value for the chi-squared test for the heterogeneity of proportions (categorical variables) or linear trend (ordinal variables).*

Table 2 presents the ratios of gross and adjusted prevalence, stratified among men and women, for the occurrence of SAH, according to the investigated factors. After the analysis had been adjusted, the prevalence of SAH proved to be associated with the rise in age group, a low level of education, living with a partner, being overweight or obese, and having at least one family member with a medical history of hypertension. As regards the socioeconomic factors, the lower level of education continued to be associated with a higher prevalence of SAH only among women. A directly proportional association was observed between the nutritional state and SAH in both sexes. Nonetheless, the prevalence of SAH was directly and significantly higher among those who had one of their parents, or both parents, and their grandparents, with a medical history of hypertension. Although family income, smoking, alcohol consumption, and the practice of physical activity did present a trend of association in the gross analysis, these were contradictory and all of them lost significance after the adjustment ( Table 2 ).

**Table 2:** – Gross or adjusted prevalence ratios (OR) of systemic arterial hypertension (SAH≥140/90 mmHg or treatment) according to demographic, socioeconomic, behavioral, nutritional state, and hereditary characteristics in the sample of male and female industry workers in the state of Rio Grande do Sul, RS, Brazil, 2006-2009. (N=20.792)

		Men (12,349)		Women (8,443)	
	Characteristics	Gross PR*	Adjusted PR†	Gross PR *	Adjusted PR †
First level	Age (years)	p<0.001	p<0.001	p<0.001	p<0.001
	18 to 29	1	1	1	1
	30 to 39	3.66 (3.01-4.45)	3.43 (2.78-4.21)	4.32 (3.35-5.56)	3.79 (2.93-4.90)
	40 to 49	7.78 (6.48-9.35)	7.13 (5.84-8.71)	11.37 (9.00-14.44)	9.85 (7.70-12.60)
	50 to 59	13.7 (11.38-16.56)	12.09 (9.81-14.90)	19.64 (15.24-25.31)	17.28 (13.29-22.47)
	Skin color	p=0.936	-	p=0.509	-
	White	1		1	
	Não White	1.01 (0.89-1.13)		0.95 (0.78-1.13)	
	Marital status	p<0.001	p=0.028	p<0.001	p<0.001
	Without partner	1	1	1	1
	With partner	2.27 (1.98-2.60)	1.17 (1.02-1.35)	1.50 (1.28-1.76)	1.28 (1.09-1.51)
	Education	p<0.001	p=0.182	p<0.001	p=0.001
	Incomplete elementary	1.67 (1.41-1.96)	1.05 (0.87-1.26)	4.00 (2.82-5.67)	1.58 (1.08-2.29)
	Complete elementary	0.91 (0.75-1.10)	0.84 (0.70-1.03)	2.10 (1.45-3.05)	1.46 (1.00-2.14)
	Complete high school	0.83 (0.69-1.00)	0.88 (0.73-1.06)	1.51 (1.02-2.21)	1.30 (0.87-1.92)
	Incomplete/complete higher education	1	1	1	1
	Monthly family income (Brazilian reals)	p=0.005	p=0.701	p=0.002	p=0.055
	≤ 800	0.83 (0.72-0.95)	0.96 (0.83-1.12)	1.50 (1.19-1.88)	1.30 (1.02-1.66)
	801 to 1,200	0.77 (0.67-0.89)	0.89 (0.77-1.03)	1.35 (1.07-1.69)	1.20 (0.95-1.51)
	1,201 to 1,800	0.78 (0.67-0.90)	0.89 (0.76-1.03)	1.40 (1.09-1.79)	1.16 (0.91-1.49)
	> 1,800	1	1	1	1
Second level	Smoking	p<0.001	p=0.262	p=0.014	p=0.133
	Non-smoker	1	1	1	1
	Ex-smoker	1.70 (1.48-1.96)	1.03 (0.91-1.18)	1.40 (1.10-1.77)	0.96 (0.77-1.20)
	Smoker	1.23 (1.08-1.40)	1.08 (0.95-1.23)	0.91 (0.71-1.16)	0.83 (0.67-1.05)
	Alcohol consumption	p=0.435	-	p=0.002	p=0.824
	Does not consume	1		1	1
	Consumes	1.04 (0.94-1.16)		0.67 (0.52-0.86)	0.98 (0.77-1.25)
	Physical Activity	p<0.001	p=0.361	p=0.323	-
	Active	1	1	1	
	Inactive	1.35 (1.20-1.51)	1.04 (0.94-1.16)	0.93 (0.80-1.08)	
	Nutritional state (BMI)	p<0.001	p<0.001	p<0.001	p<0.001
	Normal (< 25 kg/m^2^)	1	1	1	1
	Overweight (25 to 29.9 kg/m^2^)	2.78 (2.42-3.18)	1.94 (1.69-2.22)	3.34 (2.78-4.02)	2.04 (1.69-2.45)
	Obese (≥ 30 kg/m^2^)	5.43 (4.71-6.27)	3.36 (2.90-3.89)	6.76 (5.66-8.08)	3.60 (2.98-4.36)
	Presence of SAH in the family	p<0.001	p<0.001	p<0.001	p<0.001
	No	1	1	1	1
	Mother or Father	1.61 (1.44-1.80)	1.49 (1.34-1.65)	2.17 (1.82-2.58)	1.78 (1.50-2.10)
	Mother and Father	2.94 (2.50-3.46)	2.16 (1.86-2.52)	4.68 (3.81-5.76)	2.77 (2.27-3.38)
	Mother, Father, and Grandparents	3.31 (2.61-4.19)	2.95 (2.40-3.62)	4.47 (3.48-5.74)	3.22 (2.54-4.07)

* P-value for the Wald test for the heterogeneity of proportions (categorical variables) or linear trend (ordinal variables) obtained by means of Poisson regression with robust variance; † Adjusted analysis by multivariate model, including the variables with p<0.20 in the gross analysis. First Level: adjustment among the sociodemographic variables; Second Level: adjustment among the variables of the first level with p<0.20 plus the behavioral, nutritional state, and hereditary variables.

## Discussion

The present study’s findings point out that sociodemographic, nutritional, and family history factors are associated with the occurrence of SAH in industry workers. Moreover, this study also found that the prevalence of SAH increased according to the advance in age and the marital status, in both sexes. The unfavorable socioeconomic conditions also proved to be associated with the presence of SAH, especially among women.

The prevalence of SAH confirmed in this study was below that confirmed in other studies conducted with industry workers in Brazil.^8 , 13^ This variability may potentially be explained by this study’s period of data collection (2006 to 2009), by the diversity of criteria used to define the presence of SAH, in addition to the type of device and time of measurement of the SAH, making the comparisons among studies difficult.^19^ Furthermore, male workers presented a higher prevalence of SAH when compared to women, which runs in line with a study previously conducted with industry workers.^7^

Although cross-sectional studies do not allow for cause-effect relationships to be established, it is possible to consider that the physiological changes related to the advance in age can increase risks in the cardiovascular system.^1 , 20 , 21^ In this study, the prevalence of SAH was significantly higher among workers of 40 years of age and over. One similar finding was observed in a study conducted with workers from the metallurgical and steelworking sector,^13^ illustrating that workers of over 40 years of age are a priority for SAH preventive actions and intervention.

Workers who reported living with a partner presented a higher prevalence of SAH. A similar finding was previously observed, in which living with a partner was associated with other morbidities and SAH.^22^ By contrast, when analyzing the influence of the level of education on SAH, our study identified that the women with a lower level of education presented a higher probability for SAH, which was similar to previous findings in worker samples.^23 , 24^ Although studies have sought to identify differences between the potential risk factors for SAH among workers of both sexes, what stands out is that the biological susceptibility related to sex or to difference in gender is not linked to the health risks inherent to the work process for both sexes.^25^ Nevertheless, it is possible to highlight that women often occupy less prestigious positions and with lower salaries in companies,^26^ aspects that can reflect the higher prevalence of SAH in women of lower socioeconomic classes.

In the present study, the overweight and obese workers presented a two to three-fold greater probability of having SAH. Prior studies corroborate a significant association between the nutritional state and SAH, including studies conducted with workers.^12 , 20 , 27 , 28^ This finding demonstrates a possible relationship between the increase in the prevalence of SAH with the nutritional transition of the population.^6 , 28^ By contrast, as regards the family history of SAH, our study confirmed a significantly higher prevalence of SAH among workers who reported a prior family history of SAH in their father, mother, or grandparents. Previous studies on the theme demonstrate a significant importance of the hereditary burden for the development of SAH.^29 , 30^

The main positive points of this study include its sample size, including workers from different industrial sectors of the state of Rio Grande do Sul. The present study adopted a standardized method of data collection, as well as all procedures necessary for the adequate measurement of one’s blood pressure. It should also be emphasized that this study sought to explore the potential factors associated with the occurrence of SAH, considering the adoption of a multivariate analysis model. However, the findings of this study should be interpreted considering a few limitations. This study used secondary data, collected from 2006 to 2009, not including the collection of occupational variables. As this is a cross-sectional study, it may be subject to the presence of reverse causality among the investigated associations. The presence of bias on the part of a healthy worker or survivor cannot be totally discarded, since this study considered only active workers, that is, those who were working when this study was applied, excluding those who were on leave due to health problems, for example. Another limitation of this study is due to the lack of an intra- and inter-examiner variability analysis for the measurements of systolic and diastolic blood pressures. Finally, the analysis by industrial sector, as well as possible sociodemographic differences between sectors, were hindered due to the broad heterogeneity of the number of workers investigated in each sector.

Although the prevalence of SAH found in our study was relatively low, it was observed that the occurrence of SAH in industry workers is influenced by sociodemographic, nutritional, and family history factors. However, further studies are warranted and should focus on identifying potential occupational risk factors (type of occupation, work shift, time of service, presence of a food program in the workplace, among others) associated with SAH, concentrating on specific measures and actions geared toward combatting the occurrence of chronic non-communicable diseases and protecting the full wellbeing of the worker.

## Conclusions

This study’s findings, in the studied sample, pointed out that sociodemographic, nutritional, and family history factors are associated with the occurrence of SAH. It was found that the prevalence of SAH increased in accordance with advances in age and marital status, in both sexes. The unfavorable socioeconomic conditions were also associated with the presence of SAH, primarily among women. Such evidence can contribute to the formulation of effective interventions for the prevention of SAH, together with the policies geared toward worker health.
